# Pseudotumor Cerebri Following Nexplanon® Implantation

**DOI:** 10.7759/cureus.2648

**Published:** 2018-05-18

**Authors:** Benjamin E Jewett, Rick L Wallace, Olga Sarkodie

**Affiliations:** 1 Department of Obstetrics and Gynecology, Quillen College of Medicine, East Tennessee State University, Johnson City, USA; 2 Quillen College of Medicine Library, Quillen College of Medicine, East Tennessee State University, Johnson City, USA

**Keywords:** pseudotumor cerebri, nexplanon, etonogestrel, idiopathic intracranial hypertension

## Abstract

There has been much discussion about the relationship between hormonal contraceptives and the development of pseudotumor cerebri (PTC). Some have claimed that progestin-containing contraceptives, such as levonorgestrel intrauterine devices, are linked to PTC. However, the relationship and mechanism of PTC following the use of progestin-containing contraceptives remain controversial. We present a case of PTC following implantation of Nexplanon® (etonogestrel) (Merck Sharp & Dohme BV, Haarlem, The Netherlands), a progestin contraceptive. Clinicians should be vigilant for visual disturbances, headache, nausea, and other signs of increased intracranial pressure in patients receiving progestin-containing contraceptives.

## Introduction

Nexplanon® (Merck Sharp & Dohme BV, Haarlem, The Netherlands) is a long-acting, reversible, implantable etonogestrel contraceptive. Traditionally implanted in the medial arm, it has become popular due to its three-year lifespan and greater than 99% efficacy rate at preventing pregnancy. Many side effects have been documented. Common adverse reactions include oligomenorrhea (34%), headache (25%), amenorrhea (22%), menorrhagia (18%), vaginitis (15%), acne vulgaris (14%), weight gain (14%), and abdominal pain (11%) [1].

Pseudotumor cerebri (PTC), also known as benign intracranial hypertension or idiopathic intracranial hypertension, is a clinical syndrome resulting from increased intracranial pressure, causing symptoms such as headache, nausea, vomiting, visual disturbances, and papilledema. It is defined by the modified Dandy criteria, a series of diagnostic criteria which distinguish PTC from other forms of secondary intracranial hypertension [2].

There have been numerous reports of PTC in patients receiving hormonal contraceptives, but there has so far been inadequate evidence to conclusively state a definitive relationship between the two. Of note, levonorgestrel, which only differs structurally from etonogestrel by a single carbon group, has been noted by some to be associated with PTC, though this link is controversial [3]. In this paper, we present a case of PTC following Nexplanon implantation.

## Case presentation

A 27-year-old woman was referred by her ophthalmologist to our gynecologic office for evaluation after papilledema was found on her ocular examination. Upon further questioning, she complained of a subacute onset of intractable headaches, worse in the morning and aggravated by leaning forward, vision loss in her right visual field, nausea, vomiting, and balance problems. She stated that these problems began in January. The patient had a Nexplanon device implanted in November. She denied any other changes in her medical history or medication since that time, except for an unsuccessful trial of over-the-counter non-steroidal anti-inflammatory drugs in an attempt to relieve her headaches. She had minimal weight gain (3 pounds, 2 ounces) during this period. Neurologic exam was non-focal. She demonstrated a marked right visual field defect on confrontation testing.

**Figure 1:**
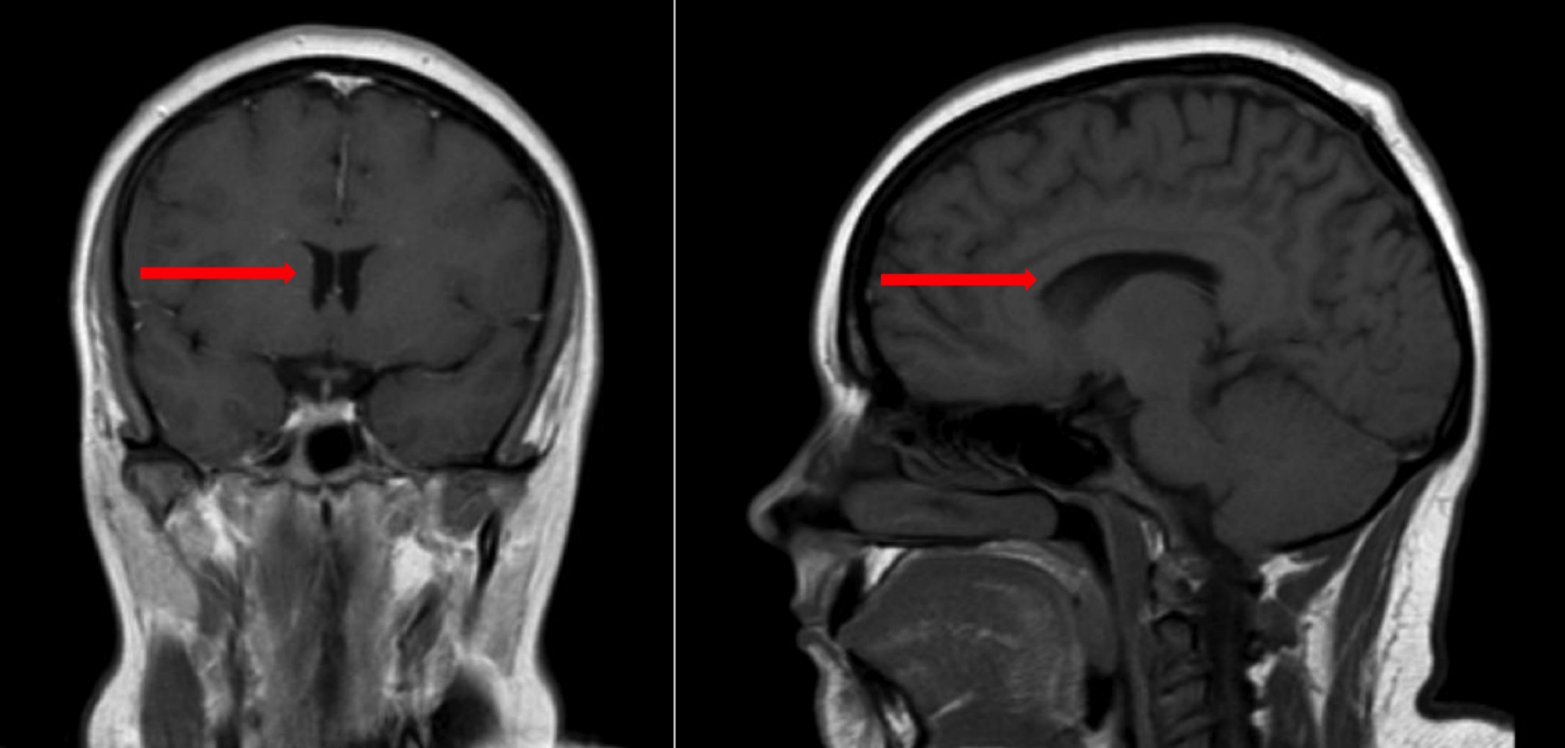
T1-weighted MRI of the Brain Sections from a normal T1-weighted magnetic resonance imaging (MRI) of the patient's brain. These images show no abnormalities that would result in secondary intracranial hypertension. Red arrows point to a normal ventricular system.

Her Nexplanon was removed in the office and she was sent to the Emergency Department for imaging and a lumbar puncture. Computerized tomography (CT) and magnetic resonance imaging (MRI) of the head were both normal. A section from the MRI imaging is shown in Figure 1. Lumbar puncture was performed and the opening pressure was 46 centimeters (cm) of water. The cerebrospinal fluid analysis was normal, and the results are noted in Table 1. Fourteen milliliters were drained during the puncture to a closing pressure of 16 cm water. The patient noted that immediately following lumbar puncture her headache improved. Within eight hours, her visual field deficit had resolved and her headache was reduced from an 8/10 intensity to a 2/10 intensity. Per modified Dandy criteria, which are outlined in Table 2, the patient was diagnosed with pseudotumor cerebri and discharged after the lumbar puncture on acetazolamide with instructions to follow-up for outpatient management. Arrangements were made to place a Paragard® intrauterine copper device (CooperSurgical, Inc., Trumbull, CT) as an alternative form of contraception.

**Table 1:** Cerebrospinal Fluid Analysis Results

	Results	Reference Range
Color	Colorless	
Appearance	Clear	
Red blood cell count (per microliter)	2	
White blood cell count (per microliter)	3	0-5
Glucose	57	40-70
Protein	34	15-45
Gram stain	No organisms seen. Rare (1+) white blood cells	
Culture	No growth	
Acid-fast bacilli culture	No growth	
Fungal culture	No growth	

**Table 2:** Modified Dandy Criteria Modified Dandy criteria for the diagnosis of pseudotumor cerebri [2]

Modified Dandy Criteria
1.) Headache, visual changes, nausea, vomiting, papilledema, or other signs and symptoms of increased intracranial pressure
2.) Neurologic exam reveals no localized findings (except for cranial nerve VI palsy)
3.) Normal neuroimaging and neurodiagnostic studies, without any observable lesion in the ventricular system. Exceptions to this are evidence of elevated cerebrospinal fluid pressure (>200 millimeters water), and findings of optic nerve sheath with filled out cerebrospinal fluid spaces, empty sella turcica, or smooth-walled non-flow-related venous sinus stenosis or collapse
4.) The patient must be alert and awake
5.) There is no other cause that could lead to increased intracranial pressure

## Discussion

The relationship between PTC and hormonal contraceptives remains controversial. Some authors suggest that there is absolutely no relationship between PTC and hormonal contraception and that any reported statistical correlations are either due to chance or confounding factors [4]. Others have tried to explain the difference by suggesting that hormonal contraceptives cause rapid weight gain, which is known to precipitate PTC [5]. Some who believe that these drugs directly cause PTC have postulated that hormones directly affect cerebrospinal fluid reabsorption via modification of epithelial transport mechanisms [6].

This controversy is amplified by the financial conflicts of interest present on both sides of the debate, as has been discussed in the literature [7-9]. Regardless of financial interests, patient safety must be paramount in any discussion. While it would be regrettable if patients decline the use of long-acting reversible contraceptives (such as Nexplanon), which are generally safe and efficacious, for fear of developing an extremely uncommon adverse reaction, it would be equally dangerous if patients and health care providers were unaware of a potentially dangerous adverse event. Finding the proper balance between these two positions is no simple task.

While there is extensive documentation of pseudotumor following other types of hormonal contraception, including the levonorgestrel (Mirena®, Bayer, Whippany, NJ) intrauterine device and oral contraceptives, there is little documentation regarding Nexplanon and pseudotumor. The only mention in the literature in regard to this is a case report of two incidences of PTC following Implanon® (Merck Sharp & Dohme BV, Haarlem, The Netherlands) (etonogestrel) implantation [5]. In this case, we present a case of PTC in a patient that had no significant weight gain in the interval between Nexplanon placement and the onset of PTC. This casts some doubt on the weight-gain theory of contraceptive-induced PTC. While it is possible that PTC coincidentally arose in our patient around the time she had Nexplanon implanted, and while it cannot be stated with certainty that a single case report provides substantial evidence that Nexplanon has a causal relationship with PTC, we believe that this report adds to a sizable body of medical knowledge that suggests a relationship between hormonal contraception and PTC is potentially possible. If more cases of this are observed, it may become warranted to add the development of PTC to the list of reported adverse effects of Nexplanon.

## Conclusions

Until it can be definitely ruled out, clinicians should be alert to possible symptoms of PTC in patients receiving hormonal contraceptives, including Nexplanon. Disturbingly, 25% of Nexplanon users develop headache, underscoring the need for vigilance. If left untreated, PTC can lead to permanent blindness. While we cannot say for certain that Nexplanon led to this episode of PTC, we would encourage all physicians to be alert for this adverse effect.
